# The ‘obesity paradox’ in patients undergoing transcatheter aortic valve implantation. A specialized narrative review

**DOI:** 10.1016/j.ahjo.2025.100695

**Published:** 2025-12-08

**Authors:** Johao Escobar, Iqra Riaz, Muzamil Khawaja, Hafeez Ul Hassan Virk, Joshua Hahn, Fu'’ad Al-Azzam, Zhen Wang, Mahboob Alam, Markus Strauss, Chayakrit Krittanawong

**Affiliations:** a Department of Internal Medicine, Mobile Infirmary Medical Center, AL, USA; b Division of Cardiology, Department of Internal Medicine, University of Texas Health/McGovern Medical School, Houston, TX, USA; c Department of Cardiology, Emory University School of Medicine, Atlanta, GA, 30322, USA; d Harrington Heart & Vascular Institute, Case Western Reserve University, University Hospitals Cleveland Medical Center, Cleveland, OH, USA; e Division of Cardiovascular Surgery, Mayo Clinic, Rochester, MN, USA; f Robert D. and Patricia E. Kern Center for the Science of Health Care Delivery, Mayo Clinic, Rochester, MN, USA; g Division of Health Care Policy and Research, Department of Health Sciences Research, Mayo Clinic, Rochester, MN, USA; h The Texas Heart Institute, Baylor College of Medicine, Houston, TX, USA; i Department of Cardiology I- Coronary and Peripheral Vascular Disease, Heart Failure Medicine, University Hospital Muenster, Cardiol, 48149, Muenster, Germany; j HumanX, Delaware, DE, USA

## Abstract

Obesity has long been identified as a noteworthy risk factor for cardiovascular diseases, including aortic stenosis. Nevertheless, an unexplained phenomenon known as the obesity paradox has arisen in the context of transcatheter aortic valve implantation (TAVI), inquiring conventional beliefs. The obesity paradox refers to better clinical outcomes observed in obese individuals undergoing TAVI. These include reduced all-cause mortality, lower rates of procedural complications, and improved long-term survival rates compared to their lean counterparts. Several theories have been proposed to explain the obesity paradox in TAVI. One of the most popular is the adipose tissue-mediated cardioprotective effect hypothesis. It is known that adipose tissue could offer both protective and harmful effects on the cardiovascular system. These effects can be linked to the adipose tissue's distribution, extension, and intrinsic biological characteristics. Obesity has been associated with adipose tissue malfunction, leading to proinflammatory and deleterious effects on the cardiovascular system. As opposed to what is believed, it is suggested that a healthy obese phenotype might be facilitated by exercise, leading to outstanding cardiovascular benefits. A healthy obese phenotype is also associated with a secretome profile that has unique adipose tissue features like adequate fat storage and formation of lipid droplets, a significant capacity for adipogenesis, minimal extracellular matrix fibrosis, potential for angiogenesis, adipocyte browning, and limited infiltration or activation of macrophages. This article is presented as a specialized narrative review, synthesizing current evidence on the relationship between obesity and outcomes in patients undergoing TAVI.

## 1 Introduction

Obesity, defined by excessive accumulation of body fat, represents a chronic and progressive health condition with far-reaching consequences [1]. The most widely accepted tool for its diagnosis is the body mass index (BMI), a height-to-weight ratio valued for its practicality and reproducibility [2,3]. A BMI ≥30 kg/m^2^ is diagnostic of obesity and further classified into class I (30–34.9 kg/m^2^), class II (35–39.9 kg/m^2^), and class III (≥40 kg/m^2^) categories [2,4]. Globally, obesity has emerged as a leading driver of morbidity and mortality, with strong links to cardiovascular disease and premature death [5,6].

Adipose tissue distribution is increasingly recognized as more clinically significant than total body mass. Subcutaneous adipose tissue (SAT) lies beneath the skin, whereas visceral adipose tissue (VAT) surrounds abdominal organs, and these compartments exert distinct metabolic effects [7]. VAT in particular has proven to be a more sensitive predictor of cardiometabolic risk compared to BMI, as it reflects the pathogenic pattern of fat deposition rather than body size alone [8,9]. Furthermore, the VAT-to-SAT ratio correlates more strongly with adverse outcomes than either BMI or VAT in isolation [10]. Waist circumference serves as a simple surrogate of visceral adiposity and provides a reliable indicator of abdominal obesity [11,12]. Importantly, individuals with a normal BMI but elevated waist circumference remain at heightened cardiometabolic risk, underscoring the limitations of BMI as a sole measure of disease risk [11].

One reliable strategy for assessing visceral fat and predicting cardiovascular and metabolic disorders is measuring the waist circumference [11]. The term abdominal **obesity describes the concentration of excess fat within** the abdominal region [12]. It is important to note that relying solely on BMI to evaluate potential health complications may not be sufficient. People who **present with a normal BMI** yet have **increased waist measurements** face a higher **risk** of health complications. **Nevertheless**, evaluating risk using both **BMI and waist circumference** may be less effective, as the two measures are closely associated [11].

Aortic stenosis (AS), most prevalent in the elderly, is characterized by narrowing of the aortic valve that restricts left ventricular outflow [13]. Its classic manifestations include exertional dyspnea, angina, and syncope [14]. Echocardiography is the diagnostic cornerstone, with a transvalvular velocity ≥ 2 m/s indicating AS. Severe disease is defined by a velocity ≥ 4 m/s or a valve area ≤ 1 cm^2^, while very severe stenosis is identified when velocity exceeds 5 m/s [15]. Aortic valve replacement (AVR) remains the only therapy proven to improve prognosis. Symptomatic patients with severe AS are clear candidates for AVR, and intervention is also recommended for asymptomatic patients with reduced ejection fraction, those scheduled for concomitant cardiac surgery, or those demonstrating rapid disease progression or abnormal stress testing [15,16].

In this context, transcatheter aortic valve implantation (TAVI) has revolutionized the management of AS, expanding from an option restricted to patients at prohibitive or high surgical risk to a standard therapy considered across the full risk spectrum. According to recent longitudinal meta-analytic evidence, TAVI and SAVR demonstrate comparable rates of all-cause mortality during the first two years following intervention. However, at five-year follow-up, TAVI is associated with a statistically significant increase in mortality compared to SAVR. These findings highlight the need for careful long-term risk-benefit assessment when considering TAVI in younger or lower-risk populations and underscore the importance of individualized patient selection based on procedural durability and comorbid profiles [18]. Four-year follow-up findings from the Evolut Low Risk trial underscore the continued efficacy of TAVI in patients with low surgical risk, revealing a 26 % relative reduction in the combined risk of death or disabling stroke when compared with SAVR. This advantage was supported by favorable valve hemodynamics in the TAVI group, which included lower transvalvular gradients and a larger effective orifice area, though at the cost of a higher incidence of permanent pacemaker implantation [19]. The NOTION trial, with 10-year follow-up, similarly reported equivalent rates of death, myocardial infarction, or stroke between the two modalities [20]. Procedural outcomes differ in important ways: TAVI reduces acute kidney injury, bleeding, and new-onset atrial fibrillation, whereas SAVR is associated with fewer cases of paravalvular regurgitation and lower pacemaker implantation rates [17]. Recent literature further emphasizes that individual factors such as comorbidities, frailty, and even genotypic variation may significantly influence post-TAVI outcomes, supporting a shift toward precision medicine approaches in patient selection and management [21].

This review summarizes current knowledge of obesity, including its prevalence, mechanisms, and health consequences, and explores its interaction with outcomes after TAVI. Special emphasis is placed on the obesity paradox, wherein obese patients undergoing TAVI may experience unexpectedly favorable outcomes, and on the potential biological and clinical mechanisms underlying this phenomenon.

### 1.1 Methods

A narrative review about the obesity paradox in patients undergoing TAVI was conducted. A comprehensive literature search was performed in PubMed, Scopus, and Web of Science for articles published from January 2010 to September 2025, using combinations of keywords “transcatheter aortic valve implantation”, “TAVI”, “surgical aortic valve replacement”, “obesity”, “obesity paradox”, “cardiovascular outcomes”, “metabolic syndrome”. Additional studies were identified by screening reference list of relevant reviews. Data were synthesized qualitatively to highlight mechanisms, clinical implications, and research gaps concerning the obesity paradox in patients undergoing TAVI.

## 2 Prevalence of obesity and aortic stenosis

Recent epidemiological surveys highlight a striking global rise in obesity. By 2022, approximately 2.5 billion adults were classified as overweight, of whom nearly 890 million fulfilled criteria for obesity (BMI ≥30 kg/m^2^), equating to 16 % of the world's adult population [[22], [23], [24]]. This burden also extends to youth, with more than 159 million children and adolescents aged 5–19 years living with obesity — a nearly four-fold increase since 1990 [23,24]. Collectively, almost one in eight people worldwide now lives with obesity [[22], [23], [24]]. Between 1990 and 2022, obesity prevalence more than doubled in adults and quadrupled in younger populations, with projections estimating that by 2035, more than half of the global population may be overweight or obese [23]. Considerable regional variation persists, with Pacific Island nations, several Middle Eastern states, and parts of the Americas reporting the highest rates, while certain high-income and middle-income regions have demonstrated slower BMI growth [23,24]. In the United States, 40.3 % of adults were obese in 2021–2023, with the highest prevalence among individuals aged 40–59 years [25]. Globally, the health consequences of excess weight are substantial: from 1990 to 2019, deaths and disability-adjusted life years (DALYs) attributable to high BMI rose by more than 100 %, accounting for 8.5 % of all global deaths and nearly 6 % of DALYs [26,27].

In alignment with these global trends, obesity now affects nearly one-third of the world's population, marking a sharp increase since 1980 [28]. Prevalence has risen across all ages and both sexes, with older adults and women disproportionately represented [29]. Socioeconomic status and ethnicity remain important modifiers, and despite slower increases in BMI in high-income and select middle-income countries, wide inter-regional variation persists [30]. Obesity is also a well-established driver of cardiovascular disease [6]. In the context of aortic stenosis (AS), approximately 1.3 % of adults aged ≥65 years develop AS, but the presence of obesity increases the likelihood of AS by nearly 81 % [31,32]. Among patients undergoing transcatheter aortic valve implantation (TAVI), reported periprocedural mortality ranges from 1.1 % to 4.2 % [[33], [34], [35], [36]].

## 3 Pathogenesis of obesity

### 3.1 Inflammatory response and hormonal contribution in obesity

Obesity is often associated with a chronic low-grade inflammatory state that contributes to the development of various medical conditions [37,38]. The adipose tissue is an intricate secretory organ known to produce and release diverse proteins called adipokines, which play a crucial role in inflammatory and immune responses [39,40]. Based on a patient's biotype, adipokines can be classified as either anti-inflammatory or pro-inflammatory cytokines. In individuals with healthy profile (lean), adipose tissue mainly releases anti-inflammatory adipokines, such as transforming growth factor-beta (TGF-beta), interleukins (IL)-10, IL-4, IL-13, IL-1 receptor antagonist (IL-1Ra), adiponectin, and apelin. On the other hand, in individuals with obesity (unhealthy profile), adipose tissue mainly secretes pro-inflammatory cytokines, such as TNFs, IL-6, resistin, visfatin, leptin, angiotensin II, and plasminogen activator inhibitor-1 [41].

**Fig. 1:**
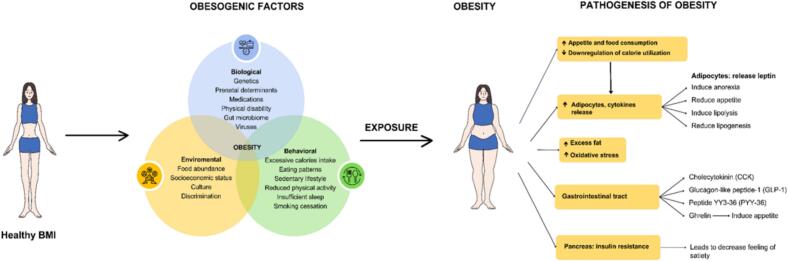
Obesogenic factors and pathogenesis of obesity.

Several hormones influence the balance between weight gain and loss (Fig. 1). Long-term appetite, energy, and body weight regulation are influenced by the inhibition of hunger through leptin [42]. Ghrelin serves as a ligand for the growth hormone (GH) secretagogue receptor and performs two essential functions: firstly, promotes the release of GH, and secondly, it enhances food intake. Before a meal, ghrelin levels in the bloodstream rise, but after food consumption and stomach distension, the levels of ghrelin decrease [43]. It is worth mentioning that elevated serum ghrelin levels have been observed following weight loss, indicating its potential contribution to the body's efforts to regain lost weight [44]. Neuropeptide Y (NPY) is a potent appetite stimulant that primarily induces the consumption of carbohydrates [45]. Studies suggest that the NPY and its receptor may significantly influence appetite regulation, feeding behavior, and even the onset of obesity. When NPY activates the NPY Y2 receptor it promotes angiogenesis, immune cells infiltration, and the proliferation of fat cells, resulting in metabolic syndrome and abdominal obesity [46].

**Fig. 2:**
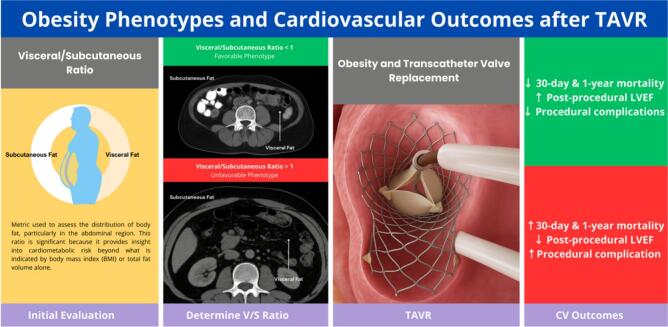
Visceral vs. subcutaneous adipose tissue and phenotypic impact on TAVI outcomes.

The combination of several obesogenic factors can lead to the development of obesity. Various changes can develop with obesity, including those that promote insulin resistance, cytokine release, oxidative stress, and the buildup of fatty tissue. As a result, appetite and food intake may increase, triggering further inflammatory and immunological responses. Over time, these changes can increase the risk of cardiovascular complications, such as myocardial infarction, stroke, valvular diseases, and even death (Fig. 2).

This infographic illustrates the contrasting effects of adipose tissue distribution on clinical outcomes in patients undergoing transcatheter aortic valve replacement (TAVR). The left panel depicts the favorable phenotype, characterized by a visceral-to-subcutaneous adipose tissue (VAT/SAT) ratio ≤ 1. Patients with this phenotype demonstrate lower cardiovascular risk, reduced mortality, and higher procedural success rates. Anatomically, this phenotype is associated with predominant subcutaneous fat distribution, anti-inflammatory adipokine profiles, and healthier metabolic states.

### 3.2 Lifestyle and environmental factors

Weight gain arises when caloric intake exceeds expenditure, with diet and physical activity as central determinants. Energy-dense foods such as fried potatoes, processed/red meats, and sweets promote obesity, whereas yogurt, fruits, nuts, vegetables, and whole grains are protective [47]. High intake of ultra-processed foods further contributes to global obesity trends [48]. Reduced physical activity and sedentary lifestyles substantially increase risk [49].

The gut microbiome also influences obesity pathogenesis: dysbiosis promotes systemic inflammation and metabolic dysregulation, suggesting a therapeutic target [50,51]. Sleep disruption is another key driver; short sleep, circadian misalignment, and night shift work are linked to obesity [52,53]. Importantly, **obstructive sleep apnea (OSA)** common in obesity induces sympathetic activation, intermittent hypoxia, and inflammation, independently predicting cardiovascular morbidity and mortality [55,56].

Although smoking cessation improves long-term health, it is often associated with weight gain, requiring tailored interventions [54].

### 3.3 Microenvironment and gut microbiome

In recent years, scientific interest has grown in understanding **the potential role of gut microbial populations in shaping the onset of obesity**. Among **people affected by obesity, the composition of the gut microenvironment often shifts, resulting in a broader spectrum of pathogenic organisms** compared with **leaner peers** [57]. This environment is more likely to produce harmful variations that can cause severe complications [58]. Available evidence points to an association between **gut microbial profiles, obesity, and the propensity for adiposity-related health complications**, including systemic inflammation and type 2 diabetes. Nevertheless, it is yet to be established whether manipulating the gut microbiome can effectively treat obesity [59].

Research further shows that **a relatively higher Prevotella-to-Bacteroides proportion** is linked to more pronounced **weight reduction** in individuals adhering to high-fiber, calorie-restricted diets [60]. Following weight loss, **the gut microbiota in those with obesity may adopt characteristics resembling that of lean individuals, frequently marked by an increased Firmicutes-to-Bacteroides ratio**; with weight regain, **this measure** often returns to its previous state [61].

### 3.4 Genetic factors

Studies have shown that 40–70 % of the variation in human obesity can be attributed to genetic factors [62,63]. Children of obese biological parents are at markedly higher risk: those with one obese parent are three to four times more likely to develop obesity, and the risk increases more than tenfold when both parents are obese [64]. Beyond genetics, parents also shape children's eating behaviors by providing both the genetic background and the shared household environment that influence dietary patterns from early life [67]. These findings highlight the importance of preventive strategies that target both family-level behaviors and genetic susceptibility.

Obesity can also result from rare genetic abnormalities such as Prader–Willi and Bardet–Biedl syndromes, as well as monogenic disorders involving the melanocortin 4 receptor, leptin, leptin receptor, or proopiomelanocortin (POMC) pathways [65]. Such monogenic and syndromic forms typically present as severe early-onset obesity and are often accompanied by additional systemic features [65,66].

## 4 Obesity and cardiovascular outcomes

### 4.1 Mortality

Higher BMI has been consistently associated with an elevated likelihood of both overall and cardiovascular mortality, particularly among individuals with class III obesity [68,69]. People with excess body weight may be considered metabolically healthy if they lack the typical cardiometabolic disturbances linked to adiposity [70]. Nonetheless, evidence suggests that individuals living with obesity face increased mortality hazards even in the absence of such metabolic abnormalities [71].

Further insights from a large-scale analysis of over 160,000 TAVI cases have shown that while obesity itself may not directly predict mortality, several obesity-related comorbidities such as renal failure requiring dialysis, liver disease, and pneumonia are strongly associated with adverse in-hospital outcomes [72]. These findings highlight the importance of careful pre-procedural evaluation in obese patients, as their higher burden of comorbidities may partially offset the survival advantage described in the obesity paradox. In the context of transcatheter aortic valve implantation (TAVI), the interplay between obesity and survival has been a focus of ongoing debate [73]. Although excess adiposity is traditionally tied to unfavorable cardiovascular outcomes through its contribution to hypertension, diabetes, and atherosclerosis [76], several large observational studies have identified an **obesity paradox**, in which overweight and mildly obese patients demonstrate comparable or occasionally lower mortality risk relative to those of normal weight [75,76].

Explanatory hypotheses for this paradox include greater energy reserves and, in some cases, enhanced muscle mass, which may improve resilience during both the intervention and recovery phases. On the other hand, severe obesity especially when accompanied by central fat accumulation remains consistently linked with poorer prognoses [76].

Analyses from registries and multicenter cohorts reveal varied results: certain datasets indicate reduced 1-year mortality among overweight and class I obese patients [73,75], while others find no meaningful association between BMI category and survival once comorbidities are accounted for [74]. Furthermore, indicators of fat distribution, such as waist-to-hip ratio or measures of visceral adiposity on imaging, often provide stronger predictive value for mortality risk than BMI alone [74,75].

### 4.2 Heart failure

The association between obesity and heart failure (HF), particularly HF with preserved ejection fraction (HFpEF), is well established [77,78]. Data from the Framingham Heart Study demonstrated that obesity confers nearly a twofold higher risk of HF compared with non-obese individuals [79]. Mechanistically, excess adiposity promotes adverse cardiac remodeling, including left ventricular hypertrophy, chamber dilation, and diastolic dysfunction, driven by expanded blood volume, increased cardiac output, and activation of neurohormonal systems such as the renin–angiotensin–aldosterone cascade. Inflammatory cytokines released by adipose tissue, including tumor necrosis factor-alpha and interleukin-6, further contribute to myocardial fibrosis and stiffness, predisposing to HFpEF [80,82].

Obesity is also implicated in the development of heart failure with reduced ejection fraction (HFrEF), often through concomitant ischemic heart disease, arrhythmias, or dilated cardiomyopathy [80,81]. Recent registry data further highlight that among TAVI patients with heart failure, HFpEF is more common than HFrEF, yet short-term in-hospital mortality does not significantly differ between the two groups. Importantly, distinct predictors of adverse outcomes exist for each phenotype, with polyvalvular disease influencing risk in HFrEF, and age, liver disease, and absence of anemia or depression impacting prognosis in HFpEF. These insights underscore the importance of phenotype-specific risk stratification in patients undergoing TAVI [84]. Interestingly, observational studies have described an “obesity paradox” in HF, where overweight and class I obese patients demonstrate better survival compared with normal-weight individuals [81,83]. This survival advantage may reflect greater metabolic reserves and reduced susceptibility to catabolic stress, though it diminishes in severe obesity, where outcomes worsen [81].

Finally, central adiposity assessed by waist circumference or imaging markers of visceral fat provides superior prognostic value over BMI, predicting mortality and HF-related readmissions in both general HF and post-TAVI cohorts [82].

### 4.3 Coronary artery disease

An epidemiologic solid evidence that establishes a link between obesity and coronary artery disease (CAD) exists [85,86]. Per Munster Heart Study (PROCAM) findings, a clear relationship exists between BMI and other risk factors contributing to CAD. The risk factors in question include age, total serum cholesterol, low-density lipoprotein (LDL) cholesterol, systolic and diastolic blood pressure, and markers of inflammation and thrombosis. Interestingly, this study revealed that the elevated risk of mortality from CAD associated with BMI was solely due to these risk factors [85] Notably, according to a meta-analysis of multiple studies, an increase of five BMI units was associated with a 29 % increase in risk for coronary heart disease [86].

### 4.4 Arrythmia and conduction disorders

Obesity is a major determinant of atrial fibrillation (AF), driven by atrial remodeling and altered conduction [87]. The ARIC study showed that nearly one in five AF cases is attributable to obesity [88]. AF is the most frequent sustained arrhythmia in patients undergoing transcatheter aortic valve implantation (TAVI) and is associated with higher complications, stroke, and reduced survival [89,94]. Each 1 kg/m^2^ rise in BMI confers a 4–5 % higher risk of AF [91].

Mechanisms include atrial enlargement, epicardial fat infiltration, fibrosis, and pro-inflammatory and neurohormonal pathways, with comorbidities such as hypertension, sleep apnea, and insulin resistance amplifying risk [90,92,93]. AF may also diminish the survival advantage sometimes reported in overweight patients, thereby modifying the obesity paradox [95]. Weight reduction, particularly targeting visceral adiposity, lowers AF burden and recurrence [96].

Obesity has been increasingly recognized as a contributing factor to a range of conduction system disturbances beyond atrial fibrillation, including sinus node dysfunction, ventricular rhythm disorders, and conduction delays largely attributed to structural alterations in the myocardium, autonomic imbalance, and shifts in electrical signaling [97]. More recently, hospital-based data have revealed that this paradoxical survival advantage may also extend to patients admitted with supraventricular tachycardias unrelated to atrial fibrillation, where elevated body weight was linked to reduced mortality despite a greater incidence of associated complications. This further illustrates the complex, and at times favorable, influence of obesity on the electrophysiological profile of cardiac patients [98].

### 4.5 Sudden cardiac death

Excess body weight, particularly central adiposity, has been consistently linked to a heightened risk of sudden cardiac death [99]. One proposed pathway involves obesity-related prolongation of the QT interval, which increases susceptibility to malignant ventricular arrhythmias, compounded by the higher prevalence of coronary artery disease in this population [101,104,105].

SCD remains one of the leading causes of cardiovascular mortality, accounting for roughly 20 % of deaths in industrialized nations [100]. Large-scale population cohorts have demonstrated a stepwise increase in SCD risk with rising body mass index (BMI) [101]. In the Nurses' Health Study, for example, women with obesity experienced more than a two-fold higher incidence of SCD compared to normal-weight peers, even after controlling for traditional cardiovascular risk factors [102].

Excess adipose tissue contributes to both structural and electrophysiological cardiac remodeling. This includes left ventricular hypertrophy, myocardial fibrosis, and conduction abnormalities that favor arrhythmogenesis [103,104]. Obesity-related systemic inflammation, autonomic imbalance, and coexisting conditions such as hypertension or obstructive sleep apnea further amplify arrhythmic vulnerability [104]. In line with these mechanisms, prolonged QT intervals and diminished heart rate variability both established markers of electrical instability occur more frequently in obese individuals [105,108].

Among patients with severe aortic stenosis undergoing transcatheter aortic valve implantation (TAVI), arrhythmic complications are a recognized clinical issue, with obesity potentially exacerbating risk due to additive myocardial remodeling and hemodynamic stress [106,107]. Importantly, central obesity measures such as waist-to-hip ratio have demonstrated stronger associations with SCD risk than BMI alone [108]. Preventive strategies emphasize structured weight reduction, improved cardiorespiratory fitness, and intensive management of cardiovascular comorbidities, all of which have been linked with favorable reductions in arrhythmic risk [109].

## 5 Obesity post TAVI management

### 5.1 Lifestyle modifications

Lifestyle modification is considered the first-line approach to obesity because it is safe, affordable, and broadly effective [18,110]. In the context of transcatheter aortic valve implantation (TAVI), weight control strategies should be carefully individualized. The “obesity paradox” observed in TAVI cohorts suggests that rather than aiming for rapid weight reduction, the emphasis should be on **gradual improvements in diet quality, functional capacity, and body composition** [115]. Evidence supports that these interventions can be introduced relatively soon after the procedure, generally within the **first 2–6 weeks**, once the patient has achieved hemodynamic stability and procedural recovery [112,116]. Initiating structured rehabilitation during this early period has been shown to improve exercise tolerance and facilitate cardiovascular recovery [112,116].

Observational studies highlight that without timely intervention, patients frequently remain sedentary and at risk of malnutrition for months after TAVI [113]. Nutritional assessments have also identified suboptimal protein intake and poor diet quality in this group, underscoring the importance of early dietitian involvement [114]. Practical strategies include portion control and caloric moderation, supported by technology-based self-monitoring tools such as mobile applications and activity trackers [110,115]. Combining aerobic and resistance exercise is particularly effective in reducing fat mass while preserving lean muscle [116], and structured programs typically recommend at least 30 min of purposeful physical activity on most days of the week [38]. Taken together, current evidence suggests that **lifestyle interventions, including weight management, should be adopted within weeks after TAVI**, ideally within the first month, to maximize cardiovascular benefit while minimizing risks of malnutrition or sarcopenia [115].

### 5.2 Medications

For patients with obesity who do not achieve adequate weight loss through lifestyle modifications, pharmacotherapy serves as a critical adjunct especially in those with cardiovascular risk or post-TAVI. Among available agents, **GLP-1 receptor agonists**, such as **semaglutide** and **liraglutide**, offer substantial weight reduction (up to 15 %) and demonstrate favorable cardiovascular safety profiles, even in high-risk cohorts [117,118]. These agents improve glycemic control, reduce appetite, and exert anti-inflammatory and vasodilatory effects relevant to cardiac health.

More recently, **dual GIP/GLP-1 receptor agonists**, particularly **tirzepatide**, have shown enhanced efficacy, with reported weight loss approaching 21 %, while maintaining cardiovascular tolerability in individuals with metabolic dysfunction [119,120]. Though data in post-TAVI populations is still evolving, their mechanism and safety profile support their potential use in this setting.

Given the cardiovascular risks associated with **sympathomimetics** and **naltrexone-bupropion**, their role in patients post-TAVI is limited. **Orlistat**, though modestly effective (~5–8 % weight loss), remains an option when gastrointestinal side effects are manageable and cardiovascular neutrality is preferred [121].

Taken together, **GLP-1 and GIP/GLP-1 therapies currently represent the cornerstone of obesity pharmacotherapy** in high-risk cardiac populations [122]. Future work should clarify their role specifically in TAVI recipients, but present data supports cautious, individualized use.

### 5.3 Bariatric surgery

Patients with a BMI ≥40 kg/m^2^ or ≥ 35 kg/m^2^ with obesity-related comorbidities who fail lifestyle or pharmacologic therapy may be candidates for bariatric surgery [74]. These procedures modify gastrointestinal anatomy to reduce caloric intake and support long-term weight loss, with outcomes varying by technique [93,94].

Although no guideline specifies timing after TAVI, recent data suggest that non-cardiac operations—including major abdominal surgery can be performed early without excess risk, provided valve function is stable. A multicenter registry and a national analysis both found no difference in perioperative outcomes whether surgery occurred within 30 days or later, with adverse events linked more to prosthetic valve performance than to elapsed time [125,126]. Perioperative guidelines therefore support an individualized, risk-based approach rather than fixed waiting intervals [127]. Antithrombotic strategy is also central: randomized and guideline evidence favor single antiplatelet therapy in many TAVI patients, simplifying surgical planning [16,128]. Beyond perioperative safety, bariatric surgery confers long-term reductions in mortality, heart failure, and myocardial infarction in obese populations, reinforcing its value in selected post-TAVI patients [130]. In practice, bariatric surgery may be considered within the first few months after TAVI once hemodynamic stability, prosthesis function, and antithrombotic management are confirmed.

### 5.4 Fecal microbiota transplantation

In recent years, fecal microbiota transplantation (FMT) has drawn increasing attention as a potential therapeutic option for obesity management [131]. Certain studies suggest that FMT from healthy donors can increase butyrate-producing bacteria and enhance insulin sensitivity, and promote a leaner metabolic profile [132,133]. However, the results are inconsistent: the TRIM trial, for instance, revealed no significant differences between FMT and placebo in terms of body weight or insulin resistance, with only a modest reduction in HbA1c observed at 12 weeks [131,134]. These outcomes underscore the need for better-defined clinical protocols and long-term data to determine FMT's role in sustainable weight loss strategies.

While these studies establish a general framework for the metabolic effects of FMT, its timing and applicability in patients undergoing transcatheter aortic valve implantation (TAVI) remains underexplored. A case report described successful post-TAVI FMT without complications, suggesting procedural compatibility and safety [135]. Supporting this, one study reported that elderly, frail individuals with complex comorbidities tolerated FMT well, which parallels the demographic profile of many post-TAVI patients [136]. Furthermore, studies by Rinott et al. emphasized that autologous FMT, collected during periods of metabolic improvement, can sustain weight loss effects over time [137]. Additionally, safety and efficacy data from Karimi et al. also supports the use of FMT in patients with variable diseases incliding metabolic syndrome and autoinflammtory conditions, further reinforcing its potential applicability in TAVI recipients [138]. Collectively, this evidence supports cautious implementation of FMT approximately 2–4 weeks post-TAVI, after initial recovery, for select patients requiring obesity-focused metabolic modulation.

## 6 Obesity paradox in transcatheter aortic valve implantion

Patients with higher body mass index undergoing TAVI often exhibit improved survival and procedural outcomes compared to their normal-weight counterparts, reinforcing the concept of an obesity paradox. Recent evidence also highlights that obesity itself may serve as a protective prognostic factor in this setting, further complicating traditional risk stratification [129].

Across contemporary cohorts and meta-analyses of patients undergoing transcatheter aortic valve implantation (TAVI), higher body mass index (BMI) categories particularly overweight and class I obesity are repeatedly linked with **lower short- and long-term mortality** compared with normal BMI, despite a greater burden of comorbidities and some procedure-related events [[139], [140], [141]]. In a meta-analysis of 99,829 TAVI patients, obesity (vs. normal BMI) associated with reduced **30-day** (OR 0.42), **1-year** (OR 0.48), and **long-term** mortality (OR 0.69); however, permanent pacemaker implantation and acute kidney injury were more frequent, and underweight status carried excess risk for major vascular complications [139]. Earlier single-center and registry data echo these patterns **1-year survival was better** in overweight/obese groups even when vascular complications were more common [140]. A second systematic review likewise concluded that overweight/obese patients had **lower mortality risk** after TAVI relative to normal BMI peers [141].

### 6.1 Body composition (muscle and fat distribution) rather than BMI alone

A leading explanation is that BMI is a **crude surrogate** for risk, while **body composition** preserved skeletal muscle and a more favorable fat distribution tracks outcomes more closely. Multiple TAVI studies using CT measures show that **sarcopenia** (e.g., low psoas muscle area) is common and **independently predicts mortality**, whereas patients with greater muscle mass fare better after TAVI ([142], [143], [144], [145]]. Beyond muscle, **adipose phenotype** matters: in morbidly obese TAVI patients, overall mortality was **similar** to non-obese after adjustment, but a visceral-to-subcutaneous fat ratio ≥ 1 identified a **high-risk phenotype** with increased 2-year mortality implicating **visceral adiposity** (rather than BMI per se) as a driver of adverse events [146]. A recent systematic review of **CT-derived adipose features** in TAVI further supports that **where fat is stored** (and its tissue characteristics) relates to outcomes [147]. Collectively, these data suggest that the “paradox” may reflect **more muscle** and **less visceral fat** in some higher-BMI patients, yielding a **risk profile** not captured by BMI thresholds alone.

### 6.2 Metabolic and physiological reserve

Older adults with higher BMI may carry **greater metabolic/nutritional reserve** to withstand peri-procedural stressors. In the meta-analysis of TAVI outcomes, patients with obesity also showed **slightly higher postoperative LVEF** compared with normal BMI—a signal (albeit small) consistent with **functional reserve** that could contribute to improved survival [139]. While adipokine/immunologic effects have been proposed in cardiovascular disease broadly, TAVI-specific evidence remains limited; current TAVI literature supports **phenotype (muscle mass and visceral fat)** as the more proximate correlate of outcomes [141,146,147].

### 6.3 Procedural trade-offs (complications vs. survival)

Despite lower mortality, higher BMI groups can experience **specific peri-procedural trade-offs**: meta-analytic estimates show **more permanent pacemaker implantation** and **acute kidney injury** in obesity, and **underweight** patients sustain **more major vascular complications** [139]. Single-center data show **higher vascular complications** among obese patients but **no increase in 30-day mortality** and **better 1-year survival** [140]. These findings indicate that some technical risks increase with body size, yet **do not negate** the aggregate survival advantage seen at follow-up.

### 6.4 Alternative explanations and bias

At least part of the paradox may reflect **methodologic artifacts** in observational data. **Collider/selection bias**—conditioning on receiving TAVI—can induce inverse associations between obesity and mortality even if none exists in the underlying population [148,149]. Importantly, quantitative analyses suggest collider bias is **not a complete explanation**, implying **residual confounding and true biological heterogeneity** likely coexist [150]. Thus, both **phenotypic factors** (muscle mass, visceral fat) and **analysis artifacts** (selection, treatment differences) should be considered when interpreting the paradox.

### 6.5 Synthesis for clinical interpretation

In TAVI, the “paradox” is most credible in **overweight and class I obesity**, and **least consistent** in **underweight** or **visceral-obese**/sarcopenic phenotypes. The **best-supported mechanistic frame** is **body composition** (muscle vs. fat distribution) rather than BMI alone. Practically, this favors **routine CT-based assessment of muscle and visceral fat indices** already available from pre-TAVI imaging to refine risk beyond BMI and to identify patients who might benefit from **prehabilitation or nutrition** interventions prior to (or after) TAVI.

## 7 Limitations and future directions

### 7.1 Methodological limitations

There remains a limited body of evidence on TAVI outcomes in patients with obesity, as most research is derived from small, observational cohorts [151]. Current evidence is further constrained by several methodological issues. Most available data arise from retrospective registries and meta-analyses, which are inherently susceptible to residual confounding despite multivariable adjustments [139]. Key determinants such as frailty, sarcopenia, nutritional status, and body composition are often unmeasured, limiting the ability to isolate the independent effect of obesity [144,157]. Moreover, heterogeneity in defining obesity complicates comparisons across studies. Most reports classify patients solely by BMI, despite evidence that visceral adiposity and sarcopenic obesity carry greater prognostic relevance than BMI alone [146,147]. Publication bias may also exaggerate the apparent obesity paradox, as smaller neutral or negative studies are less frequently reported [149]. Finally, pooled analyses often combine heterogeneous populations across centers and eras, with differences in valve type, operator expertise, and post-procedural care potentially influencing results [140].

### 7.2 Clinical limitations

Despite encouraging safety signals suggesting that TAVI can be performed in obese patients even those with morbid obesity, with complication rates largely comparable to non-obese patients [151] important clinical gaps remain. Meta-analyses and clinical trials suggest lower 30-day and one-year mortality among obese patients, as well as reduced long-term mortality compared with individuals of normal BMI [[152], [153], [154], [155]]. Yet, obesity in this setting has also been linked to higher rates of acute kidney injury and permanent pacemaker implantation, though obese patients may show more favorable post-operative LVEF than their lean counterparts [139].

In a large multicenter study of over 900 patients with morbid obesity, propensity-matched analyses demonstrated slightly higher vascular complication rates but similar all-cause mortality compared with non-obese patients [106]. Device success rates were somewhat lower, and prosthesis–patient mismatch (PPM) more frequent, although severe PPM did not predict two-year mortality [151]. Importantly, visceral adiposity—as reflected by a VAT:SAT ratio ≥ 1 was associated with higher two-year cardiovascular mortality and readmission [151].

Beyond such outcome data, major clinical uncertainties persist. Current guidelines provide no obesity-specific recommendations for timing or type of weight management interventions in TAVI patients [129]. Evidence is sparse regarding when lifestyle modification programs should be initiated after valve implantation, or the safe time interval before considering bariatric surgery or advanced therapies [158]. Similarly, the safety and efficacy of novel strategies such as fecal microbiota transplantation (FMT) for weight control remain largely unexplored in this older, comorbid population.

### 7.3 Future directions

To address the current limitations, several research priorities warrant emphasis. Large-scale prospective registries, both national and international, should incorporate standardized measures of adiposity and frailty with harmonized outcome reporting, thereby minimizing bias and enabling more reliable subgroup analyses [129]. In addition, stratified analyses by obesity phenotype are needed, with particular attention to visceral versus subcutaneous adiposity, sarcopenic obesity, and composite frailty indices. These efforts could be strengthened by leveraging CT-based body composition metrics, which are already routinely collected in most TAVI candidates [146,147].

Equally important are mechanistic studies that integrate advanced imaging modalities such as CT, MRI, or DXA with biomarkers of inflammation, metabolic reserve, and myocardial stress, to clarify the biological pathways underpinning the obesity paradox, including the contributions of sarcopenia and ectopic fat [159]. Beyond observational work, interventional trials—whether randomized or pragmatic are essential to evaluate structured rehabilitation, nutritional interventions, pharmacologic therapies such as GLP-1 receptor agonists, and even bariatric surgery in carefully selected post-TAVI patients [158,160]. These trials should define optimal timing, assess safety in the setting of anticoagulation and multimorbidity, and evaluate both traditional clinical outcomes and patient-reported measures.

There is a relative paucity of data regarding TAVI outcomes in patients with obesity. The majority of studies generally represent small sample sizes and are largely observational in nature. Notwithstanding, there are some recent data that suggests that TAVI can be successful within this specific patient population. TAVI has been found to be a safe option for patients with morbid obesity, with complication rates similar to those of non-obese patients [151]. According to recent meta-analyses and clinical trials, patients who underwent TAVI and had obesity experienced a lower mortality rate at 30 days, lower mortality at 1 year, and lower long-term mortality compared to individuals with normal weight [151,[153], [154], [155]]. However, patients with obesity have a higher risk of acute kidney injury and permanent pacemaker implantation. Interestingly, patients with obesity had a higher post-operative LVEF than patients of normal weight [139].

In one of the largest analyses to date, McInerney et al. analyzed over 900 patients with morbid obesity (defined as BMI >40 and/or BMI >35 with obesity related complications) with severe aortic stenosis undergoing TAVR in 18 tertiary centers. The investigators used propensity matching for outcome comparisons. Patients with obesity undergoing TAVI had a higher rate of major vascular complications (6.6 % versus 4.3 %) along with slightly lower rates of device success (84 % versus 88 %). All-cause mortality outcomes were similar in both groups [146].

The device's success tends to be lower in individuals with obesity, as they exhibit higher incidences of elevated mean aortic gradient and severe prosthesis-patient mismatch (PPM). It is worth noting that severe PPM does not predict 2-year mortality among the obese population [151]. Nevertheless, individuals exhibiting an obesity phenotype characterized by a VAT: SAT ratio of ≥1 are at a higher risk of all-cause and cardiovascular mortality rates and readmission within two years [151].

In the context of patients undergoing TAVI, there is a positive correlation between obesity and an increased likelihood of major vascular complications [101]. Notably, the transcarotid technique has been shown to exhibit a lower incidence of vascular complications compared to the transfemoral approach [156] in one observational study. Specifically, the authors used propensity matched controls for outcome comparisons between patients undergoing TAVI with obesity and analyzed those who underwent transcarotid (TC) and transfemoral (TF) approach. The investigators found the outcomes with TC approach were similar to the those with a TF approach. However, there were a limited number of centers comfortable with the TC approach. Furthermore, patients undergoing the TC approach had known issues with a TF approach which makes comparison within this limited subgroup less robust. Notwithstanding, a TC approach may be a viable option in patients with obesity undergoing TAVR who have limited vascular access options for TF approach.

## 8 Conclusions

In conclusion, obesity is a highly complex chronic condition that significantly affects the outcomes of several cardiovascular diseases. Emerging evidence suggests that TAVI can be a safe and effective option for patients with obesity. Notably, the obesity paradox stresses the need for further research. Understanding the subtle interplay between obesity and TAVI is crucial for optimizing patient outcomes and advancing cardiovascular care.

## CRediT authorship contribution statement

**Johao Escobar:** Writing – original draft. **Iqra Riaz:** Writing – review & editing. **Muzamil Khawaja:** Writing – review & editing. **Hafeez Ul Hassan Virk:** Writing – review & editing. **Joshua Hahn:** Writing – review & editing. **Fu'’ad Al-Azzam:** Writing – review & editing. **Zhen Wang:** Writing – review & editing. **Mahboob Alam:** Writing – review & editing. **Markus Strauss:** Writing – review & editing. **Chayakrit Krittanawong:** Writing – original draft, Writing – review & editing.

## Consent for publication

All the authors listed have approved the manuscript for publication.

## Ethical statement

No ethical statement is necessary.

## Declaration of competing interest

The authors declare that they have no known competing financial interests or personal relationships that could have appeared to influence the work reported in this paper.
